# Vitamin C and substituted phenols are specifically killing cancer cells

**DOI:** 10.1186/1878-5085-5-S1-A161

**Published:** 2014-02-11

**Authors:** John Ionescu

**Affiliations:** 1 Spezialklinik Neukirchen, Germany; Donau-University Krems, Austria

## 

Increased levels of transition metals like Fe, Ni, Cr, Hg and Pb are related to free radical generation, lipid peroxidation, DNA strand breaks and tumor growth in cellular systems. In order to evaluate the correlation to malignant growth in humans we investigated the accumulation of heavy metals in 8 healthy and 20 breast cancer biopsies by means of a standardized AAS methodology. A highly significant concentration of Fe, Ni, Cr, Zn, Cd, Hg and Pb was recorded in the cancer samples when compared with the control group (p ≤ 0.001). As previously reported by us, the high heavy metal levels found in various tumors may be used for therapeutic interventions with ascorbic acid or substituted phenolic mixtures. The autooxidation of vitamin C in the presence of heavy metals strongly increase superoxide and H2O2 concentrations at the tumor site with oxidosis and apoptosis induction.

In turn, bioactivation of phenolic compounds in the presence of heavy metals and an overexpressed NADPH- quinone oxidoreductase leads to a significant generation of superoxide and semiquinone radicals with deleterious effects for the metal rich malignant cells. Thus, the accumulation of transition metals in malignant tissue confers both ascorbate and phenolic compounds an increased tumor selectivity.

